# Targeting Ruminative Thinking in Adolescents at Risk for Depressive Relapse: Rumination-Focused Cognitive Behavior Therapy in a Pilot Randomized Controlled Trial with Resting State fMRI

**DOI:** 10.1371/journal.pone.0163952

**Published:** 2016-11-23

**Authors:** Rachel H. Jacobs, Edward R. Watkins, Amy T. Peters, Claudia G. Feldhaus, Alyssa Barba, Julie Carbray, Scott A. Langenecker

**Affiliations:** 1 Department of Psychiatry, University of Illinois at Chicago, Chicago, IL, United States of America; 2 Mood Disorders Centre, University of Exeter, Exeter, United Kingdom; National University of Defense Technology College of Mechatronic Engineering and Automation, CHINA

## Abstract

This pilot randomized control trial was designed to examine whether Rumination-Focused Cognitive Behavior Therapy (RFCBT) reduces rumination and residual depressive symptoms among adolescents with a history of Major Depressive Disorder (MDD) who are at risk for relapse. We also examined whether these changes in symptoms were associated with changes in functional connectivity of the posterior cingulate cortex (PCC), a key node in the default mode network (DMN). Thirty-three adolescents (ages 12–18) were randomized to eight weeks of RFCBT or an assessment only (AO) control. Twenty two adolescents successfully completed fMRI scans pre- and post-intervention. Adolescents were recruited from the clinic and community and met criteria for at least one previous episode of MDD and were currently in full or partial remission. An Independent Evaluator interviewed parent and child before and after the eight-week intervention. The left PCC (-5, -50, 36) seed was used to probe resting state functional connectivity of the DMN. Adolescents who received RFCBT demonstrated reduced rumination (F = -2.76, df = 112, *p* < .01, 95% CI [-4.72,-0.80]) and self-report depression across eight weeks (F = -2.58, df = 113, *p* < .01, 95% CI [-4.21, -0.94]). Youth who received RFCBT also demonstrated significant decreases in connectivity between the left PCC and the right inferior frontal gyrus (IFG) and bilateral inferior temporal gyri (ITG). Degree of change in connectivity was correlated with changes in self-report depression and rumination. These data suggest that rumination can be reduced over eight weeks and that this reduction is associated with parallel decreases in residual depressive symptoms and decreased functional connectivity of the left PCC with cognitive control nodes. These changes may enhance the ability of vulnerable youth to stay well during the transition to adulthood.

***Trial Registration*:** ClinicalTrials.gov NCT01905267

## Introduction

Major Depressive Disorder (MDD) with early onset can set youth on a trajectory towards lifelong illness and disability [1,2]. Although effective treatments for adolescent depression exist [3], it is sobering that nearly 50% of adolescents relapse in the two years following effective treatment [4]. No current psychosocial treatment effectively protects adolescents against depressive relapse [5], although recent research suggests that sequential administration of cognitive behavior therapy (CBT) following acute treatment with fluoxetine may be beneficial for relapse prevention [6]. In this particular study, there was an estimated probability of relapse of 9% in the fluoxetine + CBT group compared to 27% in the fluoxetine group by week 30. More research is needed to understand how to better protect adolescents from recurrent depression over the long term.

Rumination, a thought pattern that involves passively and repetitively dwelling on negative feelings and their causes and consequences remains elevated following remission from depression and prospectively predicts the severity and duration of subsequent depressive episodes [7,8]. The ruminative habit is also associated with a slower response to both antidepressant medication and CBT as well as a reduced likelihood of recovery [9,10]. Moreover, rumination has more recently been conceptualized as a transdiagnostic process and strong candidate mechanism in the treatment of internalizing disorders [11,12]. Limited work examines whether existing interventions can successfully reduce the maladaptive habit of rumination (for a review see [12]).

One such intervention, Rumination-Focused Cognitive Behavior Therapy (RFCBT) has been tested as an adjunctive intervention to treatment-as-usual (TAU; in this case antidepressant medication) among adults [13]. In this study, only 10% of those who received RFCBT relapsed over the course of six months compared to 53% of those in TAU, indicating that rumination is a modifiable mechanism even in the context of recurrent MDD. Despite these promising results, the potential efficacy of intervening earlier, before the onset of “treatment refractory” depression, has not yet been examined. Thus, adolescence may offer a particularly fruitful developmental window to change the cognitive mechanisms contributing to depression [14].

The technology offered by functional magnetic resonance imaging (fMRI) allows for the examination of potential neural mechanisms in the longitudinal course of MDD [15]. Indeed, investigators have used fMRI technology in hopes of understanding mechanisms of improvement in intervention studies (e.g., [16]). A growing body of research has specifically explored the neural signature of rumination both at rest and during task-based fMRI, including the induction of rumination ([17]. Perhaps the most replicated finding among adults is that rumination has been correlated with a set of regions labeled the default mode network (DMN) [18,19]. The DMN is a large-scale network which includes portions of the ventromedial prefrontal cortex (PFC) and posterior cingulate cortex (PCC), in addition to the inferior parietal lobes (IPL;[20]). The posterior cingulate cortex (PCC) has been identified as a key hub in the DMN [17,21]. Despite the emergence of research examining the DMN and rumination in relation to MDD, the number of studies examining these relations within adolescent samples is almost non-existent. This lack of research in adolescence is particularly striking given the vulnerability of this developmental period. Some research has begun to explore resting state functional connectivity (RSFC) as it relates to the construct of rumination among youth.

For example, one study examined the RSFC of the subgenual anterior cingulate cortex (sgACC) among currently depressed adolescents in relation to rumination [22]. Increasing levels of rumination were associated with decreased functional connectivity between the sgACC and regions involved in executive control (i.e., the Cognitive Control Network; CCN) including the inferior frontal gyrus (IFG) and medial frontal gyrus (MFG). We examined these relations among remitted young adults with a history of adolescent-onset MDD and found that rumination was inversely correlated with connectivity between the PCC and the right superior and the middle frontal gyri [23]. No study to date has examined the relation of rumination to RSFC of the PCC among currently remitted adolescents.

As such, the current investigation was designed to examine whether directly targeting rumination among a population of vulnerable adolescents in the remitted phase of MDD would be correlated with changes in the RSFC of the PCC. We hypothesized that RFCBT would reduce residual depression as well as reduce the habit of rumination and that these changes would be associated with changes in the RSFC of the PCC. Given the exploratory nature of the study, a data-driven approach was taken and connectivity of the PCC with the whole brain was examined.

## Materials and Methods

### Participants and procedures

Participants were adolescents (n = 33) who met full Diagnostic and Statistical Manual of Mental Disorders, 4^th^ edition [24] for MDD in the past, but who were currently in full or partial remission (rMDD) and were recruited at an urban academic medical center between May 2013 and May 2015. Eligibility was assessed using the Schedule for Affective Disorders and Schizophrenia for School-Age Children-Present and Lifetime Version (KSADS-PL [25]). Partial remission was defined as no more than three threshold symptoms of MDD subsequent to a full remission which was defined as the absence of any significant symptom for two weeks or longer. Full remission was defined as the absence of any clinically significant depressive symptoms (i.e., a score of three on any KSADS-PL MDD item was exclusionary, whereas a two—indicative of a subthreshold symptom—was not). Adolescents were not eligible if they had a Children’s Depression Rating Scale–Revised (CDRS-R) score higher than 45 or endorsed current suicidality with plan or intent [26]. Participants were allowed to continue all treatment including psychotherapy and medication; however, adolescents were required to be stabilized on medication, which was defined as taking an antidepressant (SSRI or SNRI) for a minimum of 12 weeks with no dose changes within the two weeks prior to enrollment. In addition to subthreshold or threshold MDD, exclusion criteria included: a WASI IQ [27] < 70, a primary diagnosis of another Axis I or II DSM-IV disorder other than ADHD, or psychotropic medication other than a stimulant for ADHD or SSRI/SNRI antidepressant (i.e., mood stabilizers). Due to the fMRI component of the study the following exclusions also applied: presence of metal in the body such as braces, claustrophobia, and current pregnancy. Comorbidity was not exclusionary if secondary to MDD with the exception of a lifetime history of autism, a psychotic disorder, mania, an eating disorder, or alcohol/substance abuse or dependence within the previous six months. The current study was approved by the University of Illinois at Chicago Institutional Review Board on December 6, 2012 and all adolescents signed assent with corresponding parent consent. The first participant began the study in March of 2013 and trial registration occurred in July of 2013 (delay due to investigator oversight). The current report describes results of the eight week intervention portion of the study. The overall study includes a two year longitudinal follow-up following the eight week intervention period which is currently ongoing. Date ranges for longitudinal follow up are June 2013 through June of 2017.

**Fig 1:**
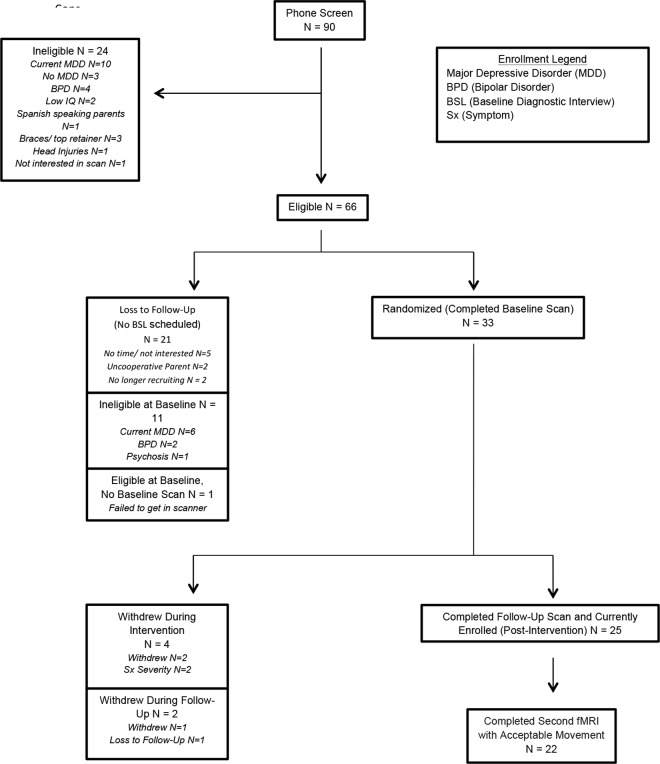
CONSORT flow diagram. MDD = Major Depressive Disorder, BPD = Bipolar Disorder.

Fig 1 illustrates the CONSORT diagram for the pilot randomized controlled trial. Thirty three participants were randomized to RFCBT (n = 17) or assessment only (AO; n = 16). Randomization was generated using Research Randomizer [28] stratified by sex and age. Participants were informed of their randomization after completing the baseline scan. Four adolescents (2 RFCBT; 2 AO) of the original 33 were withdrawn during the intervention period. Three of these were due to increasing symptoms and a need for greater outside care and one adolescent ran away from home. Twenty two of these youth (11 in each group) successfully completed a RSFC data collection pre- and post- intervention period meeting acceptable movement parameters (described below). An Independent Evaluator (IE) blind to treatment group completed baseline and eight week clinical interviews.

#### Eight-week intervention period

Adolescents randomized to RFCBT met with a therapist (first author) on a weekly basis for 45–60 minutes to receive the manualized intervention. The RFCBT intervention was adapted from materials designed for adults [13] to specifically target rumination through psychoeducation and by adopting a functional analytic approach to the learned habitual behavior of rumination [8]. The adolescent was taught to notice triggers to ruminate as well as the consequences of rumination and to shift into practicing a more adaptive strategy such as mindfulness, behavioral activation, or active problem-solving instead of ruminating. Of note, the adult version of RFCBT uses experiential processing and the identification of “flow” experiences as alternatives to rumination; however, these concepts were found to be too abstract for adolescents and mindfulness exercises were used as a form of concreteness training. Adolescents randomized to the AO group completed online questionnaires every two weeks. The family and outside clinician were notified if the adolescent’s depressive symptoms increased.

#### Reynolds Adolescent Depression Scale

The RADS (RADS; [29]) is a 30-item self-report measure of current depressive symptoms. This measure utilizes a 4-point Likert scale and has excellent internal consistency and good test-retest reliability[29]. The total score was used in the current study and higher scores reflect higher levels of depression. Adolescents completed the RADS every two weeks.

#### Ruminative Responses Scale

The 22-item RRS (RRS; [30]) was used to assess self-report ruminative tendencies every two weeks. The RRS is valid and reliable even among young adolescent populations [31].

#### Children’s Depression Rating Scale–Revised

The CDRS-R (CDRS-R; [26]) is a well validated 17-item clinician-rated depression severity measure. The reliability, validity, and sensitivity to change of the CDRS-R are well documented [32]. Inter-rater reliability (intraclass correlations, ICC = 98%) on the CDRS-R in the current study was excellent. The CDRS-R was completed at baseline and week 8 by the Independent Evaluator (IE).

#### Schedule for Affective Disorders and Schizophrenia for School-Age Children-Present and Lifetime Version

The K-SADS-PL (KSADS-PL; [25]) is a well-established structured diagnostic interview for establishing diagnoses and was completed by the IE at baseline and week eight based on interviewing both the adolescent and parent. Inter-rater reliability was good (kappa > .78).

### fMRI acquisition and preprocessing

An eyes-open resting state scan was acquired over six minutes in a 3.0 T GE Discovery scanner (Milwaukee, WI) using parallel imaging with ASSET and T2* gradient-echo axial EPI with the following parameters: 90 degree flip, field-of-view 22, matrix size = 64x64, slice thickness = 3mm, 22.2ms echo time, 44 slices, with a TR of 2000 ms for a total collection of 200 TRs. Scan parameters were adjusted following the first several scans. High-resolution anatomic T1 scans were collected across four minutes with the following parameters: 13 degree flip, field-of-view 22, matrix size 256x256, slice thickness = 1mm, 182 slices. Motion was minimized with foam pads and a cross on the display, and by conveying the importance of holding still to participants. Several steps were taken to reduce potential sources of noise and artifact. Slice timing was completed with SPM8 (http://www.fil.ion.ucl.ac.uk/spm/doc/) and motion detection algorithms were applied using FSL (http://fsl.fmrib.ox.ac.uk/fsl/fslwiki/). Coregistration of structural images to functional images was followed by spatial normalization of the coregistered T1-spgr to the Montreal Neurological Institute (MNI) template. The resulting normalization matrix was then applied to the slice-time-corrected, physiologically corrected, time series data. These normalized T2* time-series data were spatially smoothed with a 5mm Gaussian kernel resulting in T2* images with isotropic voxels, 2mm on a side. Time series were detrended and mean centered. Physiologic correction was performed by regressing out white matter and cerebral spinal fluid signals [33]. Global signal was not regressed due to collinearity violations with gray matter signal, problematic misestimates of and introductions of anticorrelations [34], and effect on distance-micromovement relationships [35]. Time-series were band-pass filtered over 0.01–0.10 Hz. Importantly, movement was addressed using regression of white matter signal as recommended in the recent literature [35,36].

#### fMRI sample and movement

All participants were trained to hold still in a mock scanner environment. In addition, to minimize head motion during the scan, foam pads were placed between the participant’s head and the coil during data acquisition. In addition, two methods were used to reduce the influence of movement on results in line with our previous work: 1) normality plots of the average standard deviation of movement values in the x, y, and z planes were examined and those with values greater than 2 standard deviations (n = 3) were excluded, 2) individuals with any TR to TR movements greater than .5mm across three consecutive TRs were also excluded (n = 2). Combining these two methods resulted in stringent criteria for movement and a sample of 22 adolescents (11 in each group). There were no significant clinical or demographic differences between the fMRI sample and the full sample of 33 randomized adolescents and randomization was not related to movement status.

#### fMRI seed-based connectivity

A 19 voxel sphere left PCC seed (–5, –50, 36, MNI coordinates) was derived based on previous work examining PCC connectivity [23,37] and previous literature examining resting state connectivity of the PCC to probe the DMN [38–40]. Correlation coefficients were calculated between mean time course for seed regions and all other voxels of the brain, resulting in 3-dimensional correlation coefficient images (r images), transformed to z scores using a Fisher transformation and compared in SPM8. Whole brain correction was achieved at *p* < .05 by using AlphaSim with 1000 Monte Carlo simulations to determine a joint threshold of height and extent (*p* < .005, cluster extent of 440 mm^3^). A full factorial second-level model including the effects of group and time was created. Given the current investigation was not adequately powered to detect treatment-by-time interactions; we chose to examine the main effect of time in the RFCBT group alone. To verify that these differences were related to treatment, we confirmed that all regions of interest (ROIs) that changed from baseline to week eight in the RFCBT group did *not* change over time in the AO group. Thus, only ROIs that changed in the RFCBT group and did not change in the AO group were evaluated.

Post-hoc analyses evaluated relations between clinical symptoms and connectivity. Two-tailed Pearson correlations between change in depression and rumination (baseline to 8 week) and change in extracted connectivity values (scan one to scan two) were conducted.

### Clinical data analyses

Mixed-effects regression models (MRMs) were conducted on the Intent-to-Treat (ITT) sample using SPSS MIXED. MRMs allow for the dependencies inherent in repeated assessments, are robust to missing data, and can be used to estimate scores using group trajectories. MRMs were used to assess the effects of treatment, and treatment-by-time (quadratic terms were included, but removed due to non-significance), on the RRS, RADS, and CDRS-R across eight weeks. Clinical results based on the sample of treatment completers as well as the fMRI sample are presented in S1 Supplement.

## Results

### Descriptive statistics

Table 1 details demographics of the full ITT sample. There were no significant differences between groups, indicating that randomization was successful. Participants were ethnically diverse and the majority of the sample was post-pubertal. Time in remission was approximately one year across groups; however, residual depression symptoms were present. Comorbid anxiety was common and present in 39% of the sample. Fifty-two percent of the sample was receiving maintenance medication treatment, 52% were engaged in psychotherapy, and 24% were taking a stimulant for comorbid ADHD.

**Table 1:** Demographics and Clinical Characteristics of Intent-to-Treat Sample.

	RFCBT (n = 17)	Assessment Only (n = 16)
	*M(SD)*	*M(SD)*
**Age**	15.41(1.97)	15.69(1.89)
**IQ Estimate**	105.13(13.78)	105.13(13.83)
**Days Since Last Episode**	469(692.25)	251(337.50)
**RADS Baseline**	64.41(12.12)	63.50(12.42)
**CDRS-R Baseline**	27.47(3.94)	28.00(6.82)
**RRS Baseline**	52.88(12.62)	50.69(12.77)
	*N(%)*	*N(%)*
**Female**	9(53%)	10(63%)
**Racial/Ethnic Minority**	9(53%)	7(44%)
**Right Handedness**	15 (88%)	14(88%)
**Post-Pubertal**^1^	8(50%)	10(67%)
**Current Comorbid Anxiety**	7(41%)	6(38%)
**Current ADHD Medication**	5(29%)	3(19%)
**Current Psychiatric Medication**	7(41%)	10(63%)
**Current Psychotherapy**	9(53%)	8(50%)

No statistically significant differences between groups.

RRS = Ruminative Responses Scale, CDRS-R = Children’s Depression Rating Scale–Revised, RADS = Reynolds Adolescent Depression Scale.

### Clinical results

**Fig 2:**
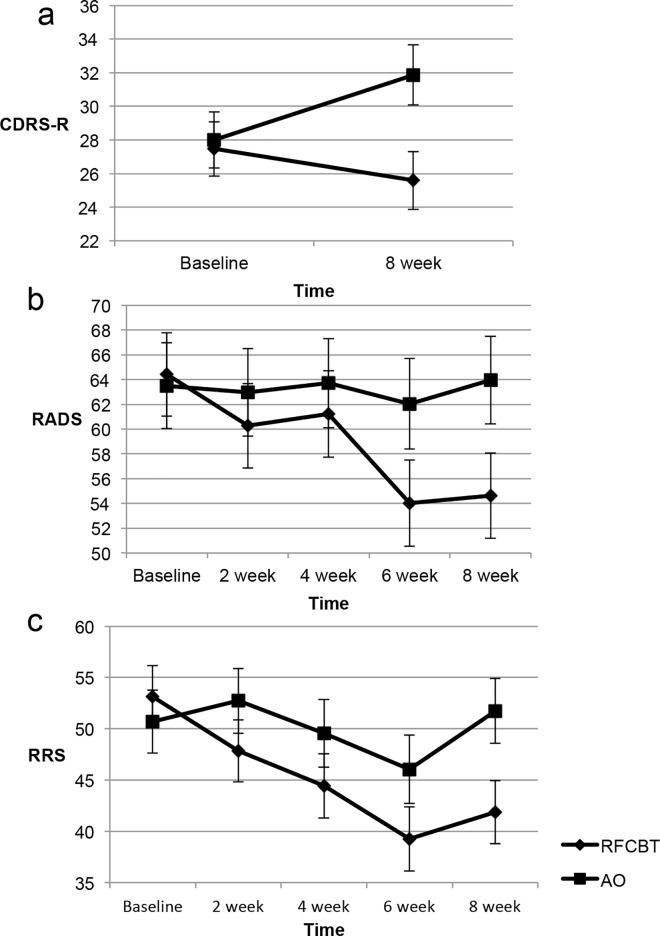
Changes in and depression and rumination over eight weeks among remitted adolescents. Predicted means and standard errors deriving from MRMs. RFCBT = Rumination-focused Cognitive Behavior Therapy, AO = Assessment Only, RRS = Ruminative Responses Scale, CDRS-R = Children’s Depression Rating Scale–Revised, RADS = Reynolds Adolescent Depression Scale. Panel a illustrates change in CDRS-R, panel b illustrates change in RADS, panel c illustrates change in RRS.

Table 2 details MRM results for the ITT sample including main and interaction effects for all outcome variables. Fig 2 depicts trajectories for predicted scores across groups. Adolescents who received RFCBT demonstrated significantly decreasing rumination (F = -2.76, df = 111.77, *p* < .01) and self-report depression across eight weeks (F = -2.58, df = 112.69, *p* < .01) relative to adolescents in the AO condition. As illustrated in Table 2, rumination and self-report depression remained constant among those in the AO group and decreased significantly (diverging by week 6) among the RFCBT group. There was a trend towards differences as measured by the IE using the CDRS-R (F = -1.44, df = 32.10, *p* = .08), wherein the AO group demonstrated a slight increase in symptoms compared to the RFCBT group who demonstrated a slight decrease. Supporting information including S1 Fig further examine clinical results.

**Table 2:** Estimates of Fixed Effects of Group, Time, and Their Interaction on Rumination and Depression.

Dependent Variable	Estimate	Standard Error	df	95% Confidence Interval	
**RRS**					
Intercept	50.46**	6.32	51.32	37.77	63.15
Treatment	.57	3.97	51.51	-7.40	8.53
Time	2.40	1.57	111.79	-.71	5.52
Treatment x Time	-2.76*	.99	111.77	-4.72	-.80
**CDRS-R**					
Intercept	28.53**	3.71	60.81	21.11	35.95
Treatment	-.53	2.32	60.81	-5.18	4.12
Time	2.40	1.26	32.14	-.16	4.97
Treatment x Time	-1.44	.79	32.10	-3.04	.17
**RADS**					
Intercept	62.49**	7.36	40.83	47.62	77.36
Treatment	.76	4.61	40.75	-8.55	10.08
Time	2.58*	1.32	112.73	-.038	5.21
Treatment x Time	-2.58**	.83	112.69	-4.21	-.94

RRS = Ruminative Responses Scale, CDRS-R = Children’s Depression Rating Scale–Revised, RADS = Reynolds Adolescent Depression Scale, df = degrees of freedom.

*p < .05

**p < .01.

### Left PCC connectivity change

**Fig 3:**
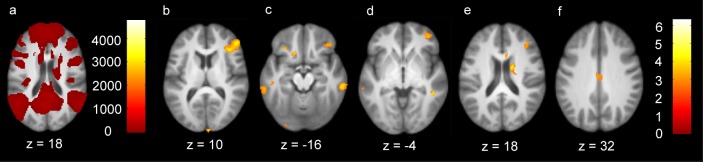
Change in connectivity of the left posterior cingulate cortex across eight weeks of Rumination-Focused Cognitive Behavior Therapy. Panel a displays significant connectivity of the left posterior cingulate seed with the whole brain among all participants at baseline. Panel b illustrates reduced connectivity with the right inferior frontal gyrus. Panel c illustrates reduced connectivity with the bilateral inferior temporal gyri. Panel d illustrates reduced connectivity with the middle frontal gyrus. Panel e illustrates reduced connectivity with the caudate body. Panel f illustrates reduced connectivity with the middle cingulate. z denotes z coordinates for axial slices and statistical maps are illustrated to the right of relevant images.

**Fig 4:**
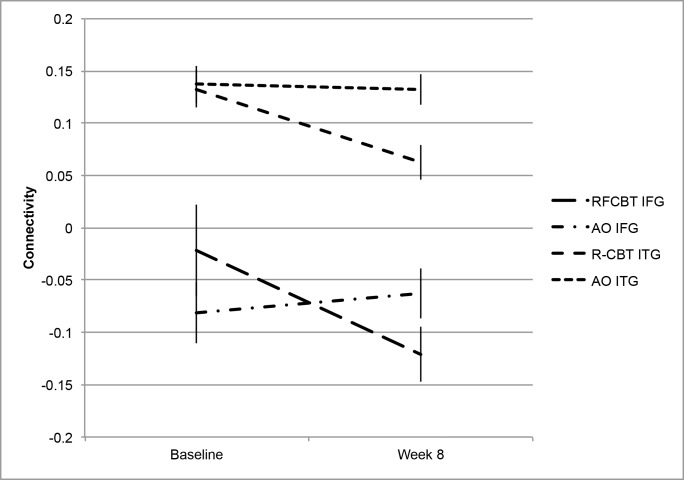
Change in connectivity across eight weeks from the left posterior cingulate seed to the right inferior frontal gyrus and right inferior temporal gyrus. Change in extracted connectivity values at baseline and week eight for the Rumination-focused CBT (RFCBT) and Assessment Only (AO groups) between the left posterior cingulate seed and right inferior frontal gyrus (IFG) and right inferior temporal gyrus (ITG).

The main effect of time in the RFCBT group revealed significant changes in several regions. Table 3 and Fig 3 detail significant change in connectivity across eight weeks within the RFCBT group. Across the intervention period, connectivity between the left PCC and frontal regions including the bilateral inferior frontal gyrus (IFG, Brodmann Areas, BA 46 and 47) decreased. Connectivity of the left PCC with medial (BA = 21) and inferior temporal (BA = 20) regions also decreased. Connectivity decreased to regions including the orbital frontal cortex, cuneus (BA = 18), and cingulate (BA = 23). Relative reductions in connectivity were also observed between the left PCC and the right caudate and mid-cingulate. Fig 4 displays extracted data between the left PCC and the right IFG and ITG. Corresponding values for the AO group are included to allow a general examination of the specificity of effects and to offer some control for the effect of time and acclimation to scanner environment.

**Table 3:** Significant Changes in whole Brain Resting State Functional Connectivity with the Left Posterior Cingulate Seed among Youth Randomized to Eight weeks of Rumination-Focused Cognitive Behavior Therapy.

**Lobe**/Region	BA	x	y	z	Z	k
**Decreasing connectivity**						
*Frontal*						
Inferior frontal	46	50	38	10	3.95	426
	47	-24	16	-24	3.75	168
	47	42	34	-16	3.08	64
	47	40	48	-4	3.15	96
Gyrus rectus	11	4	38	-26	4.81	124
*Temporal*						
Inferior temporal	20	66	-24	-22	5.23	456
Medial temporal	21	46	2	-34	3.75	157
Fusiform	20	-60	-34	-24	3.50	176
*Parietal*						
Mid- Cingulate	23	-4	-14	30	3.18	58
*Subcortical*						
Inferior Semi-Lunar		-30	-76	-48	3.68	163
		48	-70	-48	3.62	100
Caudate		6	8	14	3.42	74
		16	-2	18	3.77	72
Tuber		56	-58	-26	3.37	90
Declive		-38	-82	-20	3.20	58
*Occipital*						
Cuneus	18	4	-104	4	3.81	94
**Increasing connectivity**						
*Parietal*						
Postcentral	3	-18	-34	80	3.72	183
*Occipital*						
Fusiform	19	-28	-52	-6	3.50	68
*Subcortical*						
Cerebellum		-28	-56	-46	4.03	119
Thalamus		26	-24	2	4.22	58

All coordinates are MNI coordintes; BA = Brodmann Area; k denotes cluster size in mm^3^.

Increasing connectivity was observed from the left PCC to the postcentral (BA = 3) and fusiform (BA = 19) gyri, the thalamus, and the cerebellum.

### Clinical correlations

To reduce the number of statistical tests, correlations were run only with the two largest identified clusters: the right IFG and right ITG. Change in connectivity between the left PCC and right IFG was not significantly correlated with change in clinical measures. Change in connectivity between the left PCC and the right ITG was significantly correlated with change in self-report depression (r = .69, *p* < .01) and change in rumination (r = .48, *p* = .03).

## Discussion

This is the first study to examine whether specifically targeting rumination among adolescents in the remitted phase of MDD can minimize the ruminative habit and decrease residual symptoms of depression. This is also the first study to examine whether reducing rumination is associated with altered connectivity of the PCC, a key node of the DMN. We found that RFCBT reduced rumination which was associated with protection from increases in depressive symptoms over the course of eight weeks. Adolescents in the RFCBT group demonstrated reductions in connectivity between the left PCC to other DMN regions such as the OFC and middle cingulate as well as to regions of the CCN, such as the IFG. Importantly, decreased connectivity of the left PCC with the right ITG was correlated with reductions in rumination and depression offering a clinical context for alterations in RSFC.

Our finding that connectivity between the left PCC (a node of the DMN) and the right IFG (a node of the CCN) was reduced with RFCBT suggests that the DMN and CCN may begin to function more independently as skills for reducing rumination are learned. The IFG has been implicated in healthy emotion regulation [41] and recent work suggests it may be a key region in which neural patterns diverge between youth in the remitted stage of MDD and their healthy peers [42]. Greater connectivity between regions of the prefrontal cortex and the DMN has predicted higher levels of ruminative responding among adults [18,43] and ways in which the DMN functions in relation to task positive networks has been specifically related to rumination [19]. Thus, the current finding of decreased connectivity between the PCC and CCN regions following RFCBT suggests that the intervention may function to restore a healthier, anti-correlated pattern between the DMN and CCN. It is also possible that strategies learned in RFCBT allow for greater implicit regulation of ruminative tendencies in contrast to strategies requiring effortful control.

It is noteworthy that the caudate has been highlighted as a region that may contribute to meditative states due to its role in attentional disengagement [44]. Delivery of RFCBT to the current sample included mindfulness exercises as a form of concreteness training as well as offering a competing response for rumination. The fact that we found decreased connectivity between the PCC and the caudate suggests that self-referencing regions of the brain may be decoupling from attentional regions to reduce rumination. A growing body of work has begun to examine the neuroscience of mindfulness meditation [45] and our current findings may point to one of the mechanisms whereby mindfulness, or other attention training techniques, improve wellness–through the reduction of rumination.

Our finding that change in connectivity between the left PCC and right ITG was associated with change in depressive symptoms and change in rumination is somewhat surprising. The ITG is typically associated with semantic processing [46]; however, more recent work has begun to suggest additional functions of the ITG including self-referential processing [47]. Indeed, our current finding is consistent with a case-control RSFC study of adolescents with first-episode depression who demonstrated altered connectivity in both the PCC and ITG [48]. One additional study found greater connectivity between the amygdala and the ITG among adolescents with first-episode depression who were medication-naïve when compared to healthy controls [49]. Of note, some researchers include the medial and inferior temporal lobes as extensions of the DMN [50–52], whereas others suggest that the inferior lateral temporal lobes may be nodes within the CCN [53]. Together, these findings suggest an emerging role of connectivity of the ITG in early-onset depression. Research with larger samples of youth is needed to fully explicate the role of the ITG as it relates to depressive symptoms, response to treatment, as well as to understand how rumination is related to the ways in which the DMN and the CCN function.

Overall, the current findings build upon a recent review of fMRI studies among youth suffering from acute depression [54] that examined both task-based and RSFC studies to conclude that early-onset MDD is associated with elevated activity and connectivity in an “extended medial network” including the ventromedial and orbitofrontal cortexes (p. 209) [54]. In addition, a recent RSFC study among college students with nonclinical depressive symptoms found altered connectivity in both the bilateral ITG and IFG [55]. In addition, hyperconnectivity within the DMN and hypoconnectivity within the CCN may distinguish adults with treatment-resistant depression [56,57]. Thus, the fact that eight weeks of RFCBT was associated with a change in these abnormalities is promising. Future research can replicate these findings as well as examine whether these effects can be maintained over time.

From a theoretical perspective, the original Response Styles Theory suggests that rumination is a stable trait [58]. More recent theoretical elaboration frames rumination as a mental habit [59]. RFCBT builds upon this theoretical development to provide a treatment package that practically aids the individual in recognizing rumination and implementing healthier mental habits to protect themselves from depressive relapse. The current data suggest that among youth, the habit of rumination can be significantly reduced over the course of eight weeks. This change is associated with reductions in residual depression and also with changes in the functional connectivity of the PCC. Alternative interventions for targeting rumination have also been tested among adults [12,60] and these data support to the idea that rumination may be a modifiable mechanism in the neuropathophysiology of MDD.

The current study has several limitations. First, this was a small pilot study designed to examine feasibility, acceptability, and a preliminary test of a brain-based mechanism for a psychosocial intervention. Thus, the primary limitation of the current study is the sample size, particularly for the fMRI portion of the study. Future research adequately powered to fully examine treatment-by-time changes in connectivity is now warranted. In addition, the current sample is subject to the potential confound of ongoing psychosocial and medication treatment. Future research can evaluate the effectiveness of the intervention among a larger sample, including a population not currently receiving maintenance treatments such as psychiatric medication. A strength of the current design; however, is that it extends directly from the trial conducted with adults [13] which evaluated the efficacy of RFCBT in addition to TAU (antidepressant medication) in the treatment of medication-refractory residual depression. The fact that many adolescents across groups were receiving additional interventions, lends some support to the notion that there may be a specific and incremental gain offered from directly targeting the mechanism of rumination. On the other hand, demand characteristics may play a role in that the study and the intervention were focused on rumination and adolescents were asked to report on their level of rumination repeatedly. In addition, given the first author was the sole study therapist, further testing of the intervention with therapists blind to study hypotheses would remove potential bias. Last, it is unknown whether eight weeks of RFCBT is sufficient to delay or prevent depressive relapse among vulnerable adolescents. The current sample is currently completing longitudinal follow-up for two years, allowing for a preliminary test of this hypothesis. Last, the current study was not adequately powered to examine sex or developmental differences. The majority of youth in the current sample were post-pubertal. The effect of age and puberty on RSFC and depression course offers a rich opportunity for intervention research, particularly given the sex difference in depression that emerges during adolescence. For example, a recent examination of emotion regulation in remitted depressed and healthy children ages 8–15 found activations in portions of the IFG were positively associated with age [42]. Given our significant finding of change in connectivity between the PCC and the IFG in the current study, a larger sample should be tested to examine how the effects of age and development influence these findings.

Recurrent depressive episodes occur more frequently as illness progresses [61]; therefore, it is imperative to design interventions to protect adolescents during this critical developmental window. Indeed, effective intervention for depression among adolescents can promote long-lasting improvements in global functioning during the transition to adulthood [62]. The adolescent brain, particularly the networks supporting cognitive control, continue to develop through adolescence and into early adulthood [63]. Our findings suggest that teaching adolescents a set of skills to “get out of their head and into their life” (i.e., stop rumination) leads to both observable clinical improvement in depressive symptoms and these improvements are associated with altered patterns of network connectivity. Future research can test whether therapeutic modulation of the neural networks supporting rumination is protective of relapse over the long-term.

## Supporting Information

S1 CONSORT Checklist (DOC)

S1 Fig Reliable Change Index in the Ruminative Response Scale by Participant Engagement and Homework Completion. (TIF)

S1 Study Protocol (DOCX)

S1 Supplement (DOCX)
