# Supplementary figures and images for: Targeting Ruminative Thinking in Adolescents at Risk for Depressive Relapse: Rumination-Focused Cognitive Behavior Therapy in a Pilot Randomized Controlled Trial with Resting State fMRI
Source: PLoS One. 2016 Nov 23;11(11):e0163952. doi: 10.1371/journal.pone.0163952 (PMC5120778; doi:10.1371/journal.pone.0163952)

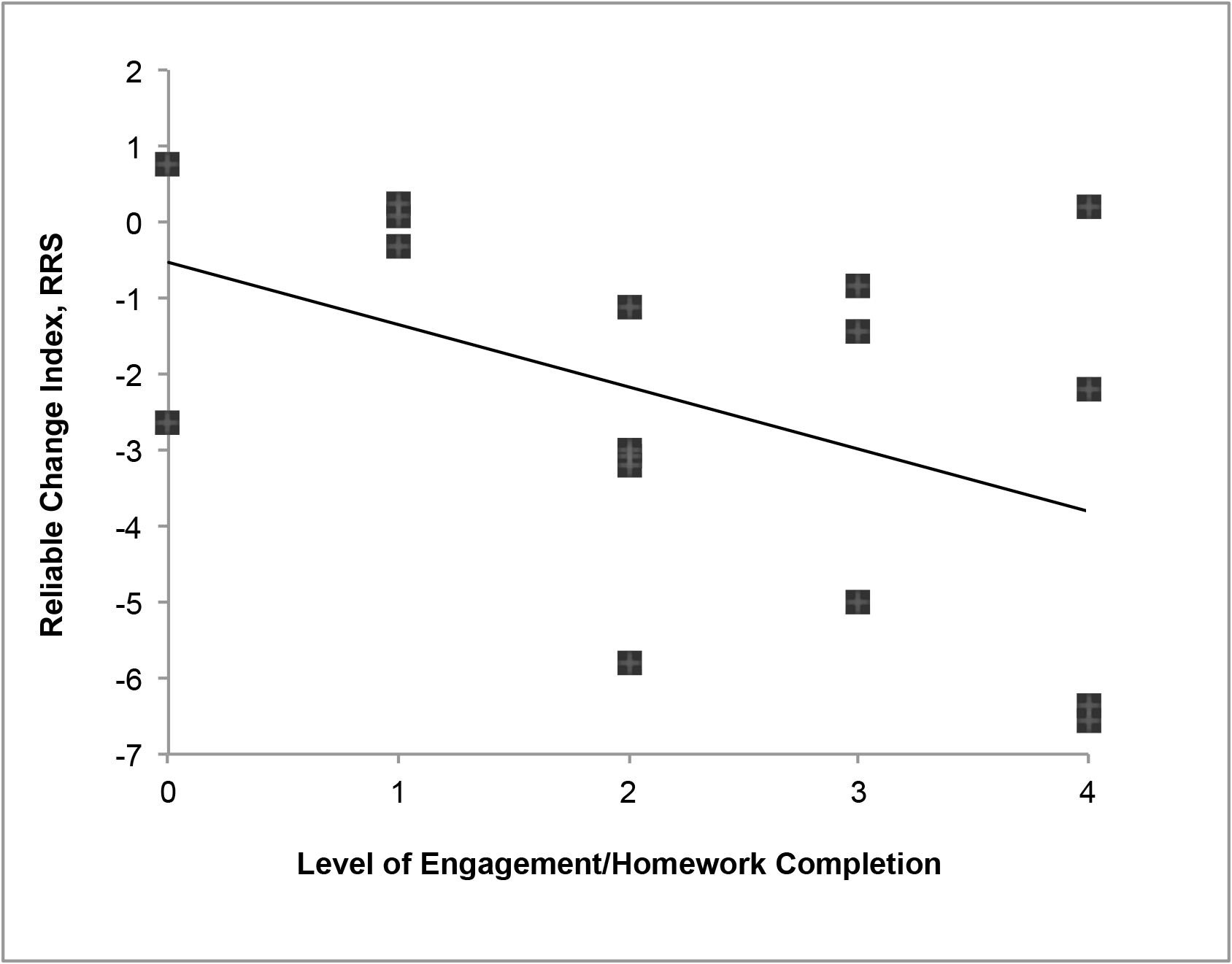

Supplement: S1 Fig — (TIF) [file pone.0163952.s002.tif]
