# Supplementary material for: Targeting Ruminative Thinking in Adolescents at Risk for Depressive Relapse: Rumination-Focused Cognitive Behavior Therapy in a Pilot Randomized Controlled Trial with Resting State fMRI
Source: PLoS One. 2016 Nov 23;11(11):e0163952. doi: 10.1371/journal.pone.0163952 (PMC5120778; doi:10.1371/journal.pone.0163952)
Supplement: S1 Study Protocol — (DOCX) [file pone.0163952.s003.docx]

Mindfulness intervention to study the neurobiology of depression: MIND

Rachel H. Jacobs

Scott A. Langenecker

University of Illinois at Chicago

Klingenstein Third Generation Fund

University of Illinois at Chicago Campus Research Board

National Center for Advancing Translational Sciences

Mind and Life Institute

IRB Protocol Version 8

08/05/15

**Specific Aims:**

Approximately 50% of adolescents with a history of depression experience recurrent symptoms within two years of recovery from a major depressive episode (Curry et al, 2011). Depression during adolescence is associated with functional impairment, suicide, and depression in adulthood (Gould et al., 1998; Lewinsohn et al., 1999). No currently available treatment protects adolescents from these lifelong sequelae. The objective of the proposed study is to examine how rumination, a maladaptive thought pattern, serves as a mechanism in the maintenance of depression, particularly in the relapse of depression among adolescents. A novel intervention targeting this mechanism, Rumination-focused Cognitive Behavior Therapy (R-CBT; Levin & Nolen-Hoeksema, 2011), adapted from Rumination-focused Cognitive Behavior Therapy for residual depression in adults (Watkins et al., in press) will be evaluated in preventing the relapse of depression among youth (ages 12 – 18) who have already experienced MDD. In addition, resting state network (RSN) connectivity analysis and functional MRI using an emotion regulation task will be used to investigate how rumination effects the functioning of the default mode network (DMN) as well as frontolimbic regions and whether R-CBT improves the functioning of this system in youth with a history of MDD.

**Aim 1:** To pilot R-CBT as a prevention program for the recurrence of adolescent depression, 30 adolescents with a previous diagnosis of MDD will be randomized to either eight weeks of R-CBT or an assessment control.

• *Hypothesis 1a:* R-CBT will result in stable or decreased ruminative tendencies; whereas youth in the control condition will demonstrate stable or increased ruminative tendencies.

• *Hypothesis 1b:* R-CBT will result in a greater decrease in residual depression symptoms compared to controls.

• *Hypothesis 1c:* Adolescents who receive R-CBT will be less likely than adolescents in the control condition to relapse to a full major depressive episode over the two year follow-up period.

**Aim 2:** To use RSN connectivity and fMRI to investigate how rumination impedes emotion regulation among adolescents with a history of MDD.

• *Hypothesis 2a:* Higher levels of rumination will be associated with hyperconnectivity of the DMN.

• *Hypothesis 2b:* Higher levels of rumination will be associated with aberrant activity in frontolimbic regions (i.e., prefrontal cortex and amygdala), during performance of a rumination induction.

• *Hypothesis 2c:* Adolescents who demonstrate reductions in rumination during R-CBT will also demonstrate normalized DMN connectivity and frontolimbic activity during a rumination induction.

**Aim 3:** To examine whether adolescents with a history of MDD differ from their healthy peers in terms of brain connectivity or functioning.

- *Hypothesis 3a*: Youth with a history of depression will demonstrate increased intrinsic functional connectivity within the DMN and between the DMN, SN, and EN relative to HC youth
- *Hypothesis 3b:* Youth with a history of depression will report higher levels of rumination when compared to HCs. Increasing levels of rumination will be associated with increased functional connectivity between the DMN and frontolimbic regions.
- *Hypothesis 3c:* When explicitly challenged with a rumination induction, youth with a history of MDD will demonstrate greater activation in DMN and frontolimbic regions in comparison to HC youth.
- *Hypothesis 3d:* Self-report rumination will predict observed connectivity within the DMN and frontolimbic regions among both youth with a history of MDD and HCs.

**Background and Significance:**

Rumination is a response to distress involving a passive and perseverative thought pattern, rather than active problem solving. A tendency to ruminate predicts the future onset, number, and duration of major depressive episodes (for a review see Nolen-Hoeksema, Wisco, & Lyubomirsky; 2008). Moreover, research has demonstrated that MDD is one of the most prevalent disorders among adolescents (Costello et al., 2006), making the availability of interventions that effectively prevent relapse critically important in ameliorating the life-long consequences of recurrent depression.

Currently, no published studies examine the impact of rumination on the neural systems implicated in emotion regulation among adolescents. The proposed study will examine whether decreases in maladaptive ruminative tendencies are associated with a normalization of brain connectivity during the resting state in regions of the DMN as well as functional activity during task requiring emotion regulation in frontolimbic regions. Disturbances in the ability to regulate emotion within frontolimbic circuits and the DMN likely contribute to the persistence of depression over the lifespan (Pizzagalli, 2011). Thus, in addition to examining how rumination disrupts effective emotion regulation, thereby contributing to the maintenance and reoccurrence of adolescent depression; this study will examine whether a rumination intervention can normalize resting state and functional disturbances in these brain regions.

Preliminary evidence suggests depression and rumination are correlated with DMN activity and connectivity. For example, greater activations in DMN regions during the resting state are associated with higher levels of depressive rumination (Hamilton et al., in press). Internally focused thought is associated with increased DMN connectivity and interferes with the performance of cognitive tasks (Sheline et al., 2010). Moreover, a ruminative self-focus is associated with enhanced recruitment of limbic, medial, and dorsolateral prefrontal regions (Cooney et al., 2010), as well as altered recruitment of mechanisms that potentiate negative affect (Ray et al., 2005). In sum, the DMN has been implicated in depression and has been linked to rumination among adults; however, these associations have not yet been explored among adolescents.

A recent review of neuroimaging studies among youth converge on hypo- and hyperactivation aberrations in brain activity in frontolimbic regions implicated in the processing and regulation of emotion (Hulvershorn et al., 2011). The emotion regulation task used in this protocol is designed to probe this aberrant frontolimbic circuitry. We hypothesize that adolescents who engage in more frequent rumination will demonstrate aberrant activations in frontolimbic regions, including increased activation in regions such as the ventrolateral prefrontal cortex (VLPFC) and dorsolateral prefrontal cortex (DLPFC) relative to adolescents who engage in less rumination. Increased connectivity between DMN regions will also be associated with rumination in adolescents with a history of MDD. We further hypothesize that R-CBT will normalize these aberrant patterns. In contrast, adolescents who do not receive R-CBT will continue to demonstrate exaggerated DMN connectivity and aberrant frontolimbic activations during emotion regulation task compared to the first scan and the R-CBT comparison group.

The proposed project is innovative in testing a novel intervention that targets a known mechanism, rumination, in the relapse of depression. This contribution will be significant in that it may curtail the high relapse rates of depression observed among adolescents. This investigation will also be the first to examine whether modulating the neural circuits involved in rumination can increase emotion regulation among adolescents, thereby preventing depression relapse.

**Research Plan:**

Participants and Protocol This study will dovetail on ongoing recruitment for the study of mood disorders among youth in the Colbeth Clinic. The clinic is located within the Institute for Juvenile Research (IJR), together with several other child psychiatry clinics and research labs, and is the home to the Pediatric Mood Disorders Program. The Psychiatry Department at UIC has the largest pediatric psychiatry academic program in Chicago for the evaluation and treatment of mood and anxiety disorders, ADHD, and autism. Chicago has a population of 11 million in the greater metro area and surrounding communities. The patient population is socio-demographically diverse, with strong representation from various racial groups, including Caucasian, African-American, and Hispanic individuals. There are few other academic programs in the city or surrounding states that are actively competing for the recruitment of affectively disordered youth. As such, the proposed project will capitalize on existing resources and infrastructure. Thirty adolescents with past MDD who are currently in partial or full remission and who agree to participate will be randomized to receive eight weeks of R-CBT in a one-on-one individual format or to an assessment only control condition.

Recruitment We will not use clinic records to identify patients. Our main source of recruitment will be flyers in the clinic and community with potential participants initiating the first contact. Examples of community sources include community centers, public libraries, health centers, and school guidance counselors. Potential participants will still be responsible for contacting study staff to initiate potential participation. Individuals will not be approached in person about the study in these community locations. Similarly, guidance counselors and school staff will only display and distribute approved study materials. They will not provide study staff with potential participant contact information. The phone screen is likely to occur over the phone, but may occur in person if the participant/participant’s family prefers. Clinicians in PMDC/Pediatrics will also give out our contact information if they identify potential participants. If a participant gives the clinician verbal consent for us to initiate contact with them, we will do so. We will also post on a variety of online recruitment sites. Examples include craigslist, clinicaltrials.gov, a listserv called the neighborhood parents network, and place an ad in the NAMI Chicago newsletter (please see attached script). Periodically, we will run advertisements on Chicago public transit (please see attached draft ad).

Randomization The randomization procedure will be generated by computer and given in a sealed envelope to the staff member meeting with the adolescent at the MRI visit. We will stratify based on gender and age (breaking at 15).

R-CBT This intervention targets rumination and other maladaptive forms of emotion regulation such as suppression and avoidance and provides skills training in effective coping strategies. Mindfulness is a key component of this intervention as a strategy for disengaging from one’s thoughts. Strategies from Dialectical Behavior Therapy (DBT), such as the use of effective interpersonal skills, are also included as methods for regulating strong emotion. R-CBT is a structured, manual based program designed to be delivered weekly over eight weeks. Sessions are 60-90 minutes in length. Twenty-five adolescents will receive eight weeks of R-CBT.

Session 1: Introduction – psychoeducation, BIG and ASK skills

Session 2: Becoming more aware – an introduction to mindfulness

Session 3: Changing the channel and behavioral activation

Session 4: Body scan, opposite action, awareness of emotion

Session 5: Psychoeducation about rumination and problem solving

Session 6: Mindfulness of breath and promoting positive emotions

Session 7: Rumination about anger and assertiveness, the context of relationships

Session 8: Mindfulness of emotions, increasing positive emotions

Assessment-only control Fifteen adolescents will complete ongoing clinical assessments as well as a pre- and post- MRI. As these adolescents will be in remission at the time of the wait-list and will receive close monitoring, this design is feasible and ethically sound. Any adolescent who experiences a relapse during the course of the study will receive a treatment referral if they do not already have a clinician.

Healthy Control Twenty-five age-, sex-, and IQ- matched healthy participants (no current or past psychiatric history) will complete clinical assessments and an fMRI scan at baseline. Healthy control participants will not receive treatment or long-term clinical monitoring.

Medication management: Participants may remain on any maintenance medication (ADHD or antidepressant SSRI/SNRI) through the course of the study. Medication will not be altered in any way because of participation in this study although psychiatrists treating participants may ask the study therapist for information regarding the participant’s symptoms for the purposes of providing the best quality of continuing care. Medication treatment is not a focus of this study, but will be closely monitored as reported by the child and parent and documented on the treatment log.

**Eligibility:**

Human Subjects Involvement and Characteristics

This study will involve 40 youth (25 R-CBT, 15 Assessment Only) ages 12-18 with a history of unipolar depression and their parent/s,and 25 matched healthy controls.

*Inclusion Criteria*

1. Previous diagnosis of Major Depressive Disorder according to DSM-IV criteria confirmed by the KSADs that is currently in partial or full remission. Partial remission is defined as not meeting full DSM-IV criteria for MDD; whereas full remission requires 8 weeks with an absence of clinically significant symptoms.

2. Stabilized on medication. All study subjects will be stabilized on medication meaning they have been on an antidepressant (SSRI or SNRI) for a minimum of 6 weeks, with no dose changes within the 2 weeks prior to entering the study. Medication changes over the course of the study will be closely documented.Participants not taking any psychiatric medication are also eligible, provided they are in remission and meet other inclusion/exclusion criteria.

3. 12-18 years of age must have one parent available who speaks English

4. Youth assent, parent consent including agreement for sessions and assessments to be recorded (digital audio recording).

5. IQ > 70, determined by a WASI scale.

*Exclusion Criteria*

1. IQ < 70

2. Currently meeting full criteria for MDD (or endorsement of current suicidality with plan or intent (assessed via KSADS and CDRS clinical interviews). Adolescents who have a CDRS score > 65 or more than 3 symptoms of MDD rated as clinically significant (scores of 3 on the KSADS) must have an outside clinician in order to participate in the study. Suicidality must be absent for a minimum of 2 weeks before entry into the study can be reconsidered.

3. Concurrent psychiatric diagnoses will not be exclusionary if secondary to MDD, with the exception of lifetime history of autism, any psychotic disorder, bipolar disorder, an eating disorder, or alcohol/substance abuse within the previous 6 months.

4. Metal braces or retainers - these subjects may enroll in the study at a later date if/when orthodontics are removed. If these potential subjects are in need of psychiatric services, they will be given referrals.

5. Claustrophobia

6. Recent (within two weeks) change in antidepressant (SSRI/SNRI) medication.

7. Severe neurological disorder or mental illness that would interfere with participation in the study.

8. Current pregnancy – Parents will be asked this information at the phone screen. At the clinic eligibility visit, female participants will be asked about sexual activity in a private room separate from their parents using the pregnancy script/screen that we have created. This script details that if a female is ineligible due to pregnancy or inadequate birth control, this information will not be disclosed to her parent(s). Participants who are sexually active will be asked about birth control method. Abstinence will be considered an approved form of contraception.

9. Psychotropic medication other than antidepressant (SSRI/SNRI) or ADHD medication.

*The same inclusion/exclusion criteria apply for healthy-control participants, with 2 exceptions: (1) healthy participants may not meet criteria for any current or past psychiatric history; (2) healthy participants may not be taking any psychiatric medication. Depressed or suicidal participants cannot be screened into the healthy cohort. All participants recruited as potential healthy controls will undergo the same clinical evaluation as participants with a history of depression. This evaluation screens for both current and past depression and suicidality, as well as for anxiety disorders, disruptive behavior disorders, psychosis, mania, and trauma. It is possible that participants who are being screened as healthy controls could meet criteria for any of these psychological disorders. If this is the case, they will be informed that they are not eligible for the study. If they meet criteria for a past history of depression, they will be informed that they could possibly participate in the randomized arm of the study; if they are interested in this option they will be consented and screened as such.

**Schedule of Assessments: Participants with a History of MDD**

**Adolescent Self-Report Assessment Schedule**

| **Measure** | **Time point** |
| --- | --- |
| RADS | Baseline, every 2 weeks over intervention, every 3 months over follow-up |
| MASC | Baseline, every 2 weeks over intervention, every 3 months over follow-up |
| RRS | Baseline, every 2 weeks over intervention, every 3 months over follow-up |
| PESQ | Baseline, week 8, 6 months, 12m, 18m, 24m |
| PANAS | Baseline, every 2 weeks over intervention, every 3 months over follow-up |
| CAMM | Baseline, week 8, 6 months, 12m, 18m, 24m |
| BADS | Baseline, week 8, 6 months, 12m, 18m, 24m |
| Metal Screen | Prior to both MRIs |
| Petersen Puberty Scale | Baseline, Year 1, Year 2 |
| Satisfaction Questionnaire | Week 8, Year 1, Year 2 |

**Parent Self-Report Assessment Schedule**

| **Measure** | **Time point** |
| --- | --- |
| BDI | Baseline, week 8, every 3 months over follow-up |
| BADS | Baseline, week 8, 6 months, 12m, 18m, 24m |
| RRS | Baseline, week 8, every 3 months over follow-up |
| Satisfaction Questionnaire | Week 8, Year 1, Year 2 |
| Demographics/History | Baseline |

**Independent Evaluator Assessment Schedule**

| **Measure** | **Time point** |
| --- | --- |
| K-SADS | Baseline, 8 week, 6 month, 12m, 18m, 24m |
| CDRS-R | Baseline, 8 week, 6 month, 12m, 18m, 24m |
| CGAS | Baseline, 8 week, 6 month, 12m, 18m, 24m |
| IE Blindness | Baseline, 8 week, 6 month, 12m, 18m, 24m |

*IE follow-up schedule may vary as an adolescent RADS self-report total score > 60 over follow-up period will trigger a full IE assessment.

*IE follow-up visits may be conducted over the phone if participants are not available to come into the clinic during the specified time period (e.g. away at college). Please see the attached instructions to the Independent Evaluator, which will be placed as a cover sheet to the interview packet when a phone assessment is necessary. In these instances, every effort will be made to schedule assessment visits during college breaks when the participant is home for holidays, but phone assessments will be used in the event this is impossible.

**Schedule of Assessments: Healthy Control Participants**

**Adolescent Self-Report Assessment Schedule**

| **Measure** | **Time point** |
| --- | --- |
| RADS | Baseline |
| MASC | Baseline |
| RRS | Baseline |
| PESQ | Baseline |
| PANAS | Baseline |
| CAMM | Baseline |
| BADS | Baseline |
| Metal Screen | Prior to MRI |
| Petersen Puberty Scale | Baseline |

**Parent Self-Report Assessment Schedule**

| **Measure** | **Time point** |
| --- | --- |
| BDI | Baseline |
| BADS | Baseline |
| RRS | Baseline |
| Demographics/History | Baseline |

**Independent Evaluator Assessment Schedule**

| **Measure** | **Time point** |
| --- | --- |
| K-SADS | Baseline |
| CDRS-R | Baseline |
| CGAS | Baseline |
| IE Blindness | Baseline |

**Description of Measures**

Kiddie Schedule for Affective Disorders and Schizophrenia for School-Age Children (K-SADS-PL; Kaufman et al., 1997). The K-SADS-PL will be administered to parents and adolescents to obtain youth diagnoses. It will also be used as a follow-up measure, by the IE, at 8-weeks, 6-month, 12-month, 18-month and 24-month visits.

Global Assessment Scale for Children (CGAS; Shaffer et al., 1983) provides a measure of global impairment and functioning over the previous month. The scale ranges from 1 (lowest) to 100 (highest) functioning. Green et al. (1994) provide evidence for the psychometric soundness of the CGAS and suggest that ratings obtained in clinical contexts may reflect evaluations of functional competence rather than symptom severity. CGAS scores will be assigned by the IE at each assessment period (baseline, 8-week, 6 month, 12-month, 18-month and 24-month).

Children’s Depression Rating Scale – Revised (CDRS-R, Poznanski & Mokros, 1996). The CDRS-R is a 17-item clinician-rated depression severity measure. Scores on the CDRS-R are based on interviews with the adolescent and parent and can range from 17 to 113, with higher scores representing more severe depression. The scale has good internal consistency (α = .85), inter-rater reliability (r = .92), test-retest reliability (r = .78) and is correlated with a range of validity indicators including global ratings and diagnoses of depression (Poznanski & Mokros, 1996). The CDRS will be administered by the IE at baseline, 8-week, 6-month, 12-month, 18-month and 24-month.

WASI The WASI is a screening instrument that estimates IQ scores rapidly and efficiently. It is a standardized, normed, and validated short form of both the WISC-III and the WAIS-III and provides a reliable and valid estimate of verbal, performance, and general intellectual functioning. The WASI is designed for use with individuals aged 6-89 years old and is individually administered. The four subtests of the WASI (vocabulary, block design, similarities, and matrix reasoning) tap various facets of intelligence, such as verbal knowledge, visual information processing, spatial and nonverbal reasoning, and crystallized and fluid intelligence. The WASI will be administered at the baseline visit.

Demographics/History. The parent will complete a demographics/history form at baseline.

Metal Screening A metal screening is delivered as part of the pre-scan procedures. This screening asks specifically about metallic implants and past experiences with metal to further ascertain any possible risks the person may incur by entering the scanner. If any metallic implants are reported that are unsuitable for the scanner, the subject is excluded from the study. If the subject is unsure of the nature of certain implants and/or there is any appreciable risk of an unknown implant (e.g., the subject had surgery for a brain aneurysm but is unaware of whether a metallic clip was placed), the experimenter will err on the side of caution and exclude the subject from the study.

Reynolds Adolescent Depression Scale – 2 (RADS; Reynolds 1986). The RADS is a 30-item self-report measure capturing constructs of depression including dysphoria, anhedonia, negative self-evaluation, and somatic complaints and has good psychometric properties (Reynolds & Mazz, 1996). The child will complete this form at baseline, every 2 weeks over intervention, and every 3 months over follow-up period.

Child and Adolescent Mindfulness Measure (CAMM; Grego, Dew, & Baer, 2005). The CAMM is a developmentally appropriate measure of mindful attention for youth with adequate internal consistency. The child will complete this form at baseline, 8 weeks, 6-month , 12-month, 18-month and 24-month evaluations.

Ruminative Responses Scale (RRS; Nolen-Hoeksema & Morrow, 1991). The RRS is a 22-item scale that captures a stable tendency to ruminate including rumination focused on symptoms as well as introspection/self-isolation and self-blame. The child will complete this form at baseline, every 2 weeks over the intervention, every 3 months over follow-up period. The parent will complete this form at baseline, week 8, and every 3 months over follow-up period.

Behavioral Activation Depression Scale (Kanter et al., 2007). This is a self-report measure designed to examine the targets of behavioral activation treatment: sleeping, activity, avoidance, rumination, social problems, work and school problems, coping skills, mood dependent behaviors, and awareness and identification of problems. The parent and child will complete this form at baseline, week 8, 6-month, 12-month, 18-month and 24-month.

Multidimensional Anxiety Scale for Children (MASC; March, Parker, Sullivan, Stallings, & Conners, 1997). The MASC is a is a 39 item 4-point Likert self-report rating scale which has shown robust psychometric properties in clinical, epidemiological and intervention studies, includes four factors, physical symptoms (tense/restless and somatic/autonomic subfactors), social anxiety (humiliation/ rejection and public performance subfactors), harm avoidance (anxious coping and perfectionism subfactors) and separation/panic anxiety. Three-week test-retest reliability for the MASC is .79 in clinical (March et al., 1997) and .88 in school-based samples (March & Sullivan, 1999). The child will complete this form at baseline, every 2 weeks over the intervention, and every 3 months over the follow-up period.

Positive And Negative Affect Scales (PANAS; Watson, Clark, & Tellegan). (1988). The PANAS includes two 10-item scales, one assessing positive affect and the other negative affect. The child will complete this form at baseline, every 2 weeks over the intervention, and every 3 months over the follow-up period.

Petersen Pubertal Developmental Scale (Petersen et al., 1988). The PDS is a widely used self-report measure of pubertal status that asks about subjective perceptions of development including changes in skin, height, pubic hair growth, and breast growth. The scale was designed for longitudinal studies and has adequate psychometric properties and good validity. The child will complete this form at baseline and Year 1.

Personal Experience Screening Questionnaire (PESQ; Winters, 1991) The PESQ provides a quick, cost-effective way to screen 12- to 18-year-olds for substance abuse. The child will complete this form at baseline, 8-weeks, 6-month, 12-months, 18-months and 24-month visits..

Beck Depression Inventory (Beck, Steer, & Brown, 1996). The Beck Depression Inventory (BDI) consists of 21 items rated on a 4-point Likert-type scale, with higher scores indicating greater depression. The parent will complete this form at baseline, week 8 and every 3-months throughout the follow-up period.

Satisfaction Questionnaire (SQ). After the 8 week intervention and at one year, parent and child rate items regarding satisfaction with the intervention.

Optional Sleep Study Measure:

Pittsburg Sleep Quality Index (Buysse, D.J., et. Al, 1989) a 19-item questionnaire that evaluates indices of sleep characteristics (e.g., sleep quality, latency, duration, efficiency) and is considered a standard measure of sleep quality (Buysse, D.J., 2004). If an adolescent and parent opts into this portion of the study, this form will be completed every night for 7 nights by the adolescent. The sleep study must take place during the 8 week intervention phase of the study.

IE Blindness: This checklist allows the independent evaluator to indicate what treatment condition they think the participant is enrolled in and how confident they are in that rating on a 4-point Likert-type scale (not all to completely certain).

**MRI Protocol for Scan 1 and Scan 2**

| **Pulse Sequence** | **Approximate Scan Time** |
| --- | --- |
| 3-Plane Localizer | 1 minute |
| Anatomical Sequence | 5 minutes |
| Resting State fMRI | 6 minutes |
| Functional Imaging  Rumination Task Self-monitoringPilot 1  Self-Monitoring Pilot 2 | 30 minutes |

MRI Scanning All adolescents with a history of MDD will be scanned twice: at baseline and then again at an 8 week follow-up time point corresponding to the end of the active intervention. All healthy control participants will be scanned once: at baseline.

Rumination Task

This event-related slow fMRI paradigm consists of rumination and distraction inductions and is based on principles of previous experimental psychology studies (i.e., Lyubomirsky & Nolen-Hoeksema, 1993). To induce negative mood, participants are asked to generate four negative life events prior to entering the scanner (Failure event, Sad family event, Hurtful event, and Frustrating event). Prior to the scan, participants will be trained on stimuli using index cards providing rumination prompts for two of the generated events. At the beginning of the ruminative thought mini-block, participants are given mood induction instructions: ‘Remember the time when someone badly hurt your feelings; Remember the saddest event in your family; Remember the time you were so sad/frustrated that you felt there was no hope for you; Remember when you failed badly at something. Use your imagination to bring this fully into your mind and picture the event.’ The instruction ‘Focus on the idea on the screen and use your imagination to think in this way’ then appears for 5 seconds. The ruminative trial includes 30 seconds of self-referential thought with prompts such as ‘what your feelings mean,’ followed immediately by 6 seconds of mood rating (‘How sad do you feel right now?’ and ‘How much are you focused on your feelings right now?’ on a 1-4 Likert scale for 3 seconds each). After every rumination event, there is a 10-15s jittered crosshair interval, followed by the distraction event. An example distraction prompt is ‘raindrops on a window.’ Prompts were modified from standard rumination protocols for a younger population (Hilt & Pollak, 2012). The entire task takes just under 10 minutes. This task has been adapted for adolescents based on fMRI research with adults (Johnson et al., 2009). After the scan is completed, a positive mood induction will be used to remedy low mood. In addition, the PI will be available to meet with any participant who may be distressed and need clinical intervention.

Self-monitoring Pilot 1 Participants will repeat the 6 minute resting state scan. At intermittent intervals of 15-45 seconds 4 brief questions will appear, probing the participant’s thinking patterns using a likert scale of 1-4. The questions will be in line with the following: 1) How much did you think about your feelings? 2) How much did your mind wander to different topics? 3) How much did you think about the past? 4) How much did you think about the future?

Self-monitoring Pilot 2 In this instance, participants will repeat the 6 minute resting state scan and a quadrant will be displayed at varying intensities throughout the 6 minutes (see attached image). The participant will be instructed to indicate the degree to which they are thinking about the past, present, or future and whether these thoughts have a negative, neutral, or positive valence. The purpose of manipulating the brightness of the quadrant over the course of the 6 minutes is to control the salience of the probe, so that it is present for the participant to note the continuous changes in their thinking pattern moment-to-moment, but not always so salient that it primes a particular response.

RSN and fMRI Data Acquisition A quadrature coil will be used to provide greater B1-field uniformity across the brain. Twenty-five axial slices will be acquired. Parameters for functional scans are: TE=25.4ms; flip angle = 82°; FOV = 20x20 cm; acquisition matrix = 64x64; TR= 1.2 sec; 4mm slice thickness with 1mm gap. High resolution anatomic images will also be acquired for co-registration and normalization of the functional data. Three dimensional inversion recovery fast spoiled gradient recalled acquisition in the steady state or 3D IRfSPGR will be obtained (TR = 13.8 ms, TE = 1.9 ms, TI= 300ms, flip angle = 25**°**, acquisition matrix = 512x192, FOV = 22x16.5 cm^2^, slices = 120, slice thickness/gap = 1.5/0 mm/mm, NEX = 1, bandwidth = ±22.7 kHz, acquisition= 4:18 min). Freesurfer will be used to reconstruct brain cortical surface from structural MRI data and co-registration. Resting state functional images will be preprocessed in SPM8 for slice timing correction, motion correction, image normalization, and 8mm Gaussian imaging smoothing. Artifact Detection Tools (ART, http://www.nitrc.org/projects/artifact_detect) software will be used for automatic detection of the global mean and motion outliers in the functional data (z-threshold = 6, and movement threshold = 2mm). A band-pass temporal filter with cutoff frequencies of .01- .10 Hz will be used on the normalized functional images to extract the low-frequency resting-state BOLD signal.

Mock scanner training, videos and CDs will be used for scanner familiarization. A color high-resolution LCD projector projects visual stimuli onto a rear projection screen, attached to the top of the magnet bore and viewed via an angled double mirror system mounted on the GE head coil.

**Optional Sleep Monitoring Task**

Procedures: All enrolled participants will have the option of completing a naturalistic sleep monitoring task. This task involves wearing a watch-like device on the wrist for eight days (7 nights) between the baseline visit and 8 week scan.

Subjects will wear actigraphs, a watch-like device, on their non-dominant wrist for eight days (7 nights) Ancoli-Israel, S. et al., 2003) The output of the Actiwatch will be digitally integrated using actigraphy principles. Actigraphy measures will be total sleep time in minutes, sleep onset latency in minutes (period between bed time and sleep onset), wake after sleep onset in minutes (time spent awake after initial onset of sleep), sleep efficiency (percentage of time in bed spent asleep), and sleep fragmentation (amount of interruption of sleep as manifested by physical movement).

In addition to wearing the device, subjects will be asked to complete two subjective sleep quality measures. Subjects will complete the Pittsburgh Sleep Quality Index (PSQI; Buysse, D.J., et al., 1989), a standard measure used to assess sleep quality. They will also complete a simple daily sleep log requiring a yes/no response for items:1) “Do you often wake up at night and have a hard time falling back to sleep again?’’, 2) ‘‘Do you have difficulty staying asleep?’’, and 3) ‘‘Do you think that you have a sleep problem?”

Participants will return the monitoring device either in person at their next study visit or by mail. Participants that fail to return the monitoring equipment, by mail or in person, will be contacted via telephone by study staff and asked to return the device. They will be informed that the device contains personal data that is unlikely to be traceable to them, but we would like to remove. If participants do not respond to these requests and they have signed a release of information to communicate with their treating clinician, we will contact their clinician so that study staff can discuss returning the monitoring device at the participants’ next appointment.

Compensation: Participants will receive an additional $15 dollars for completing the optional sleep study.

Actigraphy Data Collection & Analyses: Data will be downloaded to a secure computer and processed subject by subject with the Actiwatch software (Actiware 5.59). Data will be carefully reviewed and checked for missing values and outliers. Sleep parameters will automatically be scored using the manufacturer’s software, with an epoch length of 15 seconds and a medium wake threshold value of 40 seconds. A sleep analysis will be conducted with the sensitivity of the algorithm set to “medium” to estimate actigraphic sleep parameters. Resulting data will be further analyzed with SPSS for Windows (version 18.0; SPSS Inc., Chicago, Illinois). To examine associations between actigraphy and subjective measures of sleep difficulties and mood in patient samples, we will conduct a series of Pearson Correlations. We will also conduct a series of multivariable regression models to examine the relative contributor of sociodemographic, behavioral, and clinical predictors (including measures of sleep) to participant outcomes. The significance level in SPSS will be set at 0.05.

Clinical Risk: There is little foreseeable risk to participating in objective and subjective sleep assessment measures. Actigraph, commonly worn on the wrist like a watch, uses an accelerometer to measure movement during activity and rest. It is an objective, non-invasive measure endorsed by the American Academy of Sleep Medicine for assessing sleep patterns in community studies. Self-report assessments comprise “yes/no” responses on a 3-item daily sleep log, and the Pittsburg Sleep Quality Index is a 19-item questionnaire that evaluates sleep characteristics (e.g., sleep quality, latency, duration, efficiency). These two measures are considered standard measures of sleep quality, brief to complete, and are unlikely to cause any undue burden to participants.

**Human Subjects:**

Clinical Trials Risks The primary clinical risk is that adolescents will experience an increase of MDD or suicidality during the study. This risk is addressed through the close monitoring of symptoms every two weeks via self report. Clinically significant scores on self-report measures of depression or endorsement of critical suicidality items (see Appedndix I Suicidality Guidelines)will trigger a full assessment by the study team. Participants in the assessment only control condition will complete the same assessment schedule and an in-person or phone assessment will be triggered by clinically significant self-report or any report of suicidality. Note that we will request and highly encourage participants who endorse suicidal ideation to complete an in-person assessment. Only if the participant refuses or if there is no other option, will the suicidality assessment be completed over the phone. Participants will have 24-hour access to the research team and will receive clinical referrals if the adolescent experiences an onset of a full major depressive episode. The team will facilitate the process of depressed adolescents receiving treatment in a prompt fashion and will stay in close contact with the adolescent and family during this transition time (minimum contact once per week).

The primary risk during this study is that participants will experience an increase in depressive symptoms. This is a risk of their disease course and not a risk of the study per se. We estimate that 50% of those in the assessment only group will relapse (Curry et al., 2011), whereas approximately 10% will relapse in the R-CBT condition, consistent with a recent trial in adults (Watkins et al., 2011). The frequency of monitoring will provide clinical safety within the context of the current protocol. Families will be given feedback and treatment recommendations. If a participant develops criteria for MDD (five symptoms that are clinically significant) the participant will be given referrals for active, acute treatment outside of the study protocol if they don’t already have an (out of research) clinician. A RADS score >77 at any assessment point will trigger an interview with study staff to assess depression symptomatology. The cut-off score of 77 on the RADS to trigger safety follow-up was selected based on guidelines in the RADS professional manual which identify 77 as the threshold for clinically significant depression (Reynolds, 1987, p. 6). In addition, youth who have a CDRS-R score > 65 and more than 3 clinically significant symptoms of MDD must have an out of study primary clinician and be receiving treatment. Frequency of visits to outside providers will be documented on the outside treatment log and recorded at every in-clinic visit.

Youth will report symptoms of depression and anxiety every 2 weeks during the 8 week intervention. The RADS will serve as a trigger for a full assessment with study staff if the adolescent reports increasing or significant levels of depression (which will be monitored by comparing scores over time and a significant level of depression is defined as > 77 on the RADS). Community referrals will be provided in the event a family does not have a primary clinician.

To offset the increased risk of including adolescents who are in partial, as opposed to full remission, it is required that adolescents who have a CDRS score > 65 or more than 3 symptoms of MDD rated as clinically significant on the K-SADS (score of 3), have a non-study clinician with whom they meet regularly in order to participate in the study. The American Academy of Child and Adolescent Psychiatry practice parameters for preventing relapse and recurrence in adolescent depression recommend meeting with a clinician approximately once/month (AACAP, 2007). Thus, should participants meet this criterion for depressive symptom severity, study staff will strongly recommend treatment at this frequency. Whether the participant undergoes treatment at this rate will be at the discretion of the participant and their treating clinician. In this sense, adolescents in partial remission will be receiving treatment as usual with their non-study providers and through the research study may be randomized to receive additional intervention specifically targeting residual depression symptoms. Study staff will document on the treatment log how often participants are seeing their provider.

If intervention sessions are doubled up (2 intervention session in 1 visit), this does not represent an increased risk, as adolescents will continue to complete questionnaires regarding mood every two weeks online similar to the assessment only control. Study personnel and the Principal Investigator will continue to be available 24/7 to subjects who may need assistance with increasing depressive symptoms or emergent suicidality. The same procedures will be followed with healthy control participants should they develop depression or suicidality between the screening and baseline visits.

Consent Procedures On the first day of the study, a member of the study team will review the consent forms for participation with participant and legal guardian. The nature of the research protocol, including its potential risks and benefits, will be explained verbally to all potential subjects and their parents. To ensure that they have understood the risks and benefits, both the subject and, if under 18, the parent will be asked independently to restate in their own words the known risks and benefits of the procedure. Parents will be asked to read and sign a consent form, and their children will be asked to read and sign an assent form that has been approved by the University of Illinois at Chicago Investigational Review Boards.

Confidentiality All information obtained from the subjects is coded by number and kept in locked files. The information is not accessible to anyone but the study investigators. The research chart will be kept separate from any clinical chart that the participant may have at the IJR clinics. Some of the forms will be filled out using an online survey website, Qualtrics or REDCap. Subjects are not required to fill out the forms online, we are simply offering this for convenience. If they choose to do so, they will be given secure links to an ID-specific questionnaire – names will not be used anywhere. All results will be stored on the Qualtrics or REDCap website, but people will need a username and password to access these results. Only investigators who need to access this information for research purposes will be given this username and password. Parents and child will have separate links and will not be able to access one another’s data. In addition, given the integrated nature of the clinic and research activities, we will inform participants that if they have a clinician at IJR, the clinician will be told they are participating in the study. However, further information (other than the instance of safety concerns) will not be given to the clinician unless the participant/family authorizes this contact with a release of information form. If an adolescent reports clinically significant suicidality, both their clinician and their parent(s) will be notified immediately, but no later than 24 hours later. Furthermore, we will inform participant’s family members that if child abuse or neglect is suspected we are required to make an anonymous call to the Department of Child and Family Services (DCFS) and describe the situation as fully as possible. If the DCFS suggests that the situation requires formal reporting, then a report will be made, and DCFS will provide intervention from that point. If a report is not warranted, a further assessment of child safety and wellbeing will be made. If any risk to the child is suspected and DCFS dictates that they need not be involved, the PI will consult with the adolescent and parent to negotiate a safety plan, which will require the involvement of significant others as deemed safe and appropriate. Any needed additional support and services will be provided to the participant.

Contact with Clinician Given the integrated nature of the IJR/UIC clinic, clinicians are likely to be able to deduce if their patients are participating in research. It is explicit in consent/assent/HIPAA forms that the family must give authorization for clinical data to be shared with their clinician. However, we do not require authorization to notify the participant’s physician of safety issues such as suicidality. We will ask the participant and their family to inform us if their clinician changes over the course of the study. In this instance, they will need to complete a new authorization form.

HIPAA In addition, an authorization form to Access PHI under the Health Insurance Portability and Accountability Act (HIPAA) will be presented to parents or caregivers (the legal guardian) of adolescents under the age of 18, and to individuals directly who are 18, to access PHI.

MRI Scanning Risks *Acoustic Noise* The acoustic noise levels perceived by human subjects when undergoing MRI examination in our 3.0 Tesla magnet will be operated to ensure that human subjects no more noise than is recommended by the FDA. In addition, all participants will use disposable earplugs. *Time-Varying Magnetic Fields* The concern about the time-varying magnetic fields used in MRI is that these can, in some instances, induce stimulation of peripheral nerves, thereby producing sensations such as “twitching” or “tingling”. Gradients used in our 3.0 Tesla MRI system will typically be operated at levels below those considered to be negligible according to FDA guidelines. MR technologists receive special training to prevent peripheral nerve stimulation. *Specific Absorption Rate (SAR)* MRI scanning induces some heating of body tissues. Specific absorption rate (SAR), which determines heating, is the amount of radiofrequency (RF) energy deposited per unit volume of tissue per unit time. Our scanner console calculates SAR based on the subject’s body weight before running any pulse sequence and prohibits running of the sequence if that SAR exceeds the FDA-approved limit. Security is also provided by a restricted access area. *Static Magnetic Fields* The FDA has deemed that systems operating at 8.0 Tesla or less do not pose a significant risk. *Possible Incidental Risks* The physical confinement and isolation produced by the scanner could cause mild to moderate emotional distress, although in our extensive past experience, subjects generally tolerated the procedures remarkably well. All subjects will be able to communicate directly with technologists and study staff to inform them of any emotional or physical distress during the scanning procedure. If they wish, the scan will be terminated immediately and the subject will be removed from the scanner. *Risk Associated with Repeat Scanning* The effects of exposure are non-cumulative (i.e., exposure on multiple occasions is no greater than exposure on a single occasion). *Clinical MRI Issues* If evidence of clinical abnormalities arises during image processing, this information will be communicated to the PI who will consult with Dr. Phan (who has many years of expertise with both clinical and research neuroimaging data). If there is reason for concern, the participant and/or parents will be notified if any clinically significant lesions are detected. *Risk of Low Mood:* It is possible that subjects will experience residual low mood following the rumination induction task or in response to the overall emotional nature of the tasks. To offset this risk, a positive mood induction will be conducted outside of the scanner and the PI will be on call to deal with any emergent clinical issues.

Risk/Benefit Ratio Participants in the clinical trial may benefit from the increased monitoring of their depressive symptoms or from the R-CBT intervention. The findings of the MRI are unlikely to be of direct benefit to any of the subjects. Subjects will be informed of any clinically significant findings from the MRI or clinical assessment procedures. The subjects will receive monetary remuneration over the course of the study in the amount of $495. It is believed that the risks of participating in this study are outweighed by the protection offered by repeated assessment and clinical oversight as well as the potential benefit of improved understanding of the prevention of depression relapse.

All participants will be closely monitored through assessments throughout the course of the intervention phase as well as the follow-up. It is expected that a portion of the sample will develop increasing depressive symptoms over the course of the study. These symptoms will be closely monitored and families will be given feedback and treatment recommendations. If a participant develops criteria for full MDD (five symptoms that are clinically significant) the participant will be given referrals for active, acute treatment outside of the study protocol if they do not already have an outside clinician. We will seek to have participants continue in their assigned arm (R-CBT or assessment only), and will document outside treatment. If families wish, they may withdraw from the study all together. Any change in eligibility will be documented in the research chart using the eligibility checklist.

Referral Process The project director, Rachel Jacobs, will maintain a referral database. When notified by a P.I. or project staff that an adolescent is depressed or suicidal; Dr. Jacobs will contact the family immediately to connect them with a resource. This referral database will include low fee and sliding scale options. If an adolescent is actively suicidal, the family will be directed to a local Emergency Room. Dr. Jacobs or the Study Coordinator will check in on the family at least once a week to determine if they have established contact with a provider. There are two situations which will trigger IE or study therapist evaluation with a CDRS, as well as parent and outside clinician contact. These two situations are if a participant scores >77 on the RADS or endorses ‘sometimes’ or ‘most of the time’ on the suicidality question (#14) on the RADS. Project staff will contact families a minimum of once a week until they have linked with the appropriate services to ensure that they are not left on a waitlist. Frequency of visits to outside providers will be documented on the outside treatment log and recorded at every in-clinic visit.

End of intervention procedures Ethical principles require that all participants be given recommendations for any indicated further intervention and appropriate referrals at the point they exit intervention if necessary, (i.e., a premature termination, onset of MDD). In the last intervention session, the therapist will a) provide the family a chance to state any concerns or questions they have; b) provide a summary of progress and outcomes; c) discuss follow-up interventions; and d) make recommendations about any necessary intervention.

All end-of-intervention recommendations will be coded for data entry. Families will be given a list of possible providers for the recommended intervention and will be told that a clinical report could be sent with the youth and/or parents' authorization to any new intervention provider(s). Similar procedures will be used for premature termination at any point in the study.

Premature Termination At any time during the study, subjects may experience increased symptoms or develop clinical crises that lead to a recommendation to terminate from the assigned intervention (but almost never the assessment) portion of the protocol. For example, a subject who became depressed, suicidal, or manic and required hospitalization would almost by definition require open clinical intervention. Such subjects will be considered "premature terminators." Others will continue in study interventions, but will also require intervention outside the study as determined by the Principal Investigator. "Drop Outs": Subjects who terminate prematurely are to be distinguished from "drop outs" who are defined as subjects who refuse to furnish further data. Subjects who drop out are not eligible for further study intervention.

Any changes in eligibility status will be documented in the participant's research chart.

Recording Procedures All intervention sessions and study assessments will be recorded throughout the course of the trial. Parent and child will be required to agree to the taping of sessions and assessments to be allowed into the trial. Parents and youth who do not agree to taping procedures at the time of consent/assent will be provided with referrals outside the study. Recordings will be labeled by ID number and full names will not be used during the sessions or assessments. All session recordings will be kept in password protected digital files and access on site will be limited to study personnel. Originals of a randomly selected percentage (approximately 25%) of intervention sessions will be reviewed. These recordings will not be transcribed and they will be destroyed within one year of study completion.

Compensation Remitted MDD Adolescents [articipants will receive $50 for their diagnostic interview to confirm eligibility. For those in the R-CBT group, the participant will be reimbursed at a rate of $5 per session to offset travel expenses (5x8 = 40) and will receive parking permits as needed. Those in the assessment only group will receive $10 for completing questionnaires every 2 weeks (10x4 = 40). This $40 will be received as cash when the 8 week diagnostic interview is completed. Adolescents will receive $50 for the first MRI scan, and $75 for the second MRI scan and follow-up diagnostic interview. If all portions of the 8-week study are completed, this is a total of $215 compensation. The adolescent will directly receive cash payments in person (excluding R-CBT travel reimbursement, which will go to parents) after completing the appropriate study payment forms. The rest of the payments will arrive by check and will be addressed to the parent.

For the follow-up, the adolescent will be reimbursed $50 for the 6, 12, 18, and 24 month clinical interviews for a total of $200 over the course of 2 years. The adolescent will receive $10 for completing the 3 month self-report measures and $20 for 9 month self-report measures. Participants will receive $25 each for both the 15- and 21-month self-report measures. The total compensation for the follow-up is $280. The optional sleep study will be compensated with an additional $15. Please see the payment schedule sheet for further details.

Healthy control participants will receive $50 for the clinical screening visit and $50 for the fMRI scan.

The compensation for clinical interviews will be received via cash and for the questionnaires, participants will receive a check.

Data Plan A completely deidentified database will be created within one year of study completion, at which point all private identifiers, including contact information, will be destroyed. Audio recordings will also be destroyed one year after study completion.

Optional Sleep Study:

An optional sleep study as a multiple-PI effort has been funded by the UIC Campus Research Board. The purpose of this study is to collect feasibility data on collecting sleep data from participants across the lifespan. Participants do not need to complete the sleep study to participate in the rest of the MIND study. This is an optional add-on.

**Appendix A:** Study Procedures Flow Chart


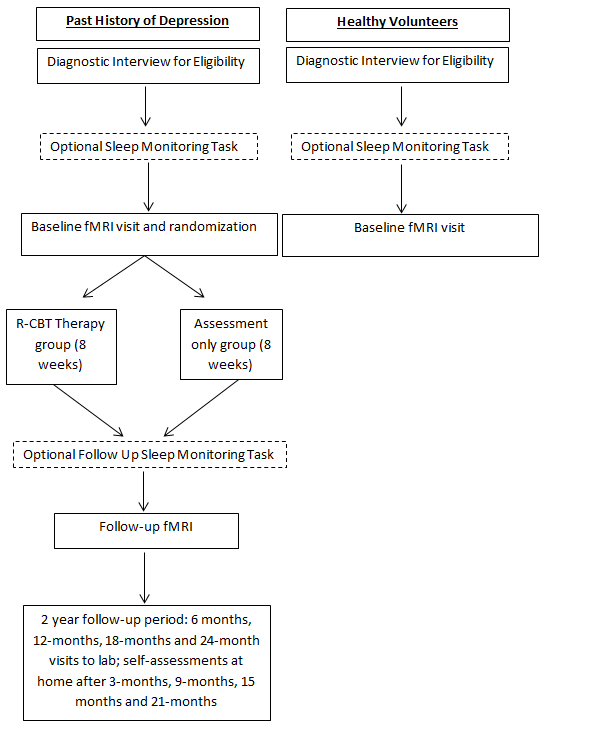


**Appendix B.** Study Eligibility Flow for Psychiatric History


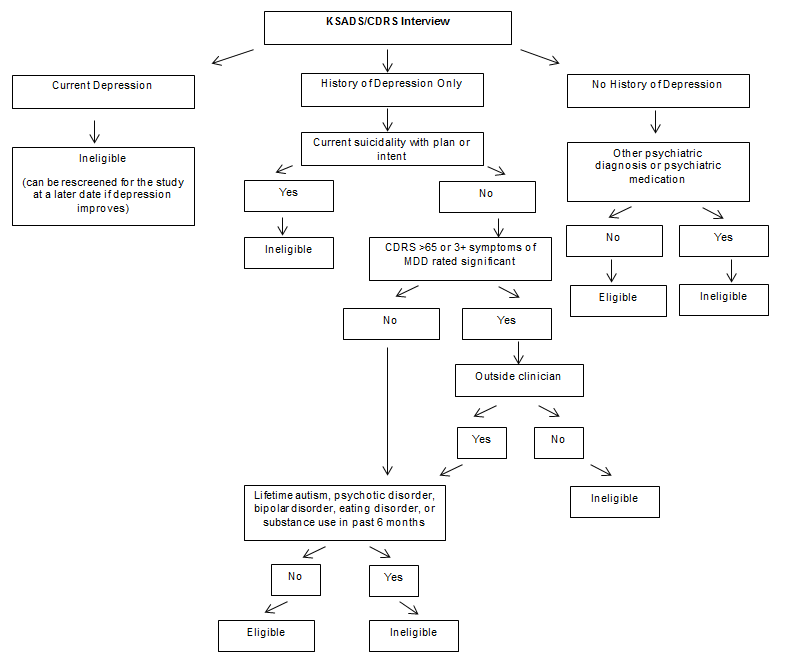


*Note: Depression and suicidality are assessed according to gold standard assessment parameters for evaluation and diagnosis of depression (AACAP, 2007). Screening interviews are conducted using the K-SADS-PL diagnostic interview with both the parent and child, separately. Both the parent and child are asked the same questions, and the independent evaluator makes a combined summary rating based on both sources of information. In the case of discrepancies in the report of depression or suicidality, the most frequent disagreements occur in the items dealing with subjective phenomena where the parent does not know, but the child is very definite about the presence or absence of certain symptoms. This is particularly true for items like guilt, hopelessness, interrupted sleep, hallucinations, and suicidal ideation. In these instances, consistent with practice recommendations (AACAP, 2007), the child’s report is weighed more heavily in determining summary ratings. If the disagreements relate to observable behavior (e.g. appetite, psychomotor agitation/slowing), the examiner queries the parent(s) and child about the discrepant information. The evaluator’s combined ratings are used to ultimately yield a diagnosis of depression. In the case of safety monitoring for continued eligibility, monitoring is triggered by the child’s report on questionnaires, but the follow up evaluation is conducted with both the parent(s) and child in the same manner described above (using a similarly formatted rating scale for current symptoms, the Children’s Depression Rating Scale).
