# Supplementary material for: Targeting Ruminative Thinking in Adolescents at Risk for Depressive Relapse: Rumination-Focused Cognitive Behavior Therapy in a Pilot Randomized Controlled Trial with Resting State fMRI
Source: PLoS One. 2016 Nov 23;11(11):e0163952. doi: 10.1371/journal.pone.0163952 (PMC5120778; doi:10.1371/journal.pone.0163952)
Supplement: S1 Supplement — (DOCX) [file pone.0163952.s004.docx]

Supplement 1

*Outside Treatment Changes across Intervention Period.* Ten adolescents experienced a change in outside treatment during the eight week intervention period (4 = RFCBT and 6 AO). Changes included tapering down or discontinuing psychotherapy (n = 3), stopping medication (n = 2), increasing SSRI (n = 3), new ADHD medication (n = 1), change in ADHD medication ( n = 1).

*RFCBT Competence and Adherence*. Twenty percent of RFCBT sessions were reviewed by two independent raters using Watkins’ (2011) competence rating scale for RFCBT. The following domains were rated: agenda setting, feedback, understanding, interpersonal effectiveness, collaboration, pacing, guided discovery, functional analysis, RFCBT principles of change, application of RFCBT techniques, and homework. Across raters and sessions the clinician received average ratings of good to very good.

*Clinical Results for Completers and fMRI Sample.* To assess these relations among the treatment completers only, a general linear model (GLM) examined changes in the primary outcomes among a sample of 15 RFCBT and 14 AO adolescents. Results among completers only parallel Intent-to-Treat (ITT) results. Self-reported depression decreased among youth randomized to RFCBT and remained stable among those in AO (F = 6.61, df = 1, *p* = .02, *η^2^*= 0.20). Similarly, adolescents randomized to RFCBT demonstrated a decrease in rumination, whereas youth in AO reported stable rumination, but this difference was only a trend (F = 3.92, df = 1, *p* = .06, *η^2^*= .13). Groups did not differ in change on the CDRS-R (F = 2.61, df = 1, *p* = .12, *η^2^* = .09). Four adolescents in the AO group relapsed and met full K-SADS-PL criteria for MDD over the course of the 8 weeks, whereas no adolescents in RFCBT relapsed (29% versus 0%).

Among the fMRI completers (11 RFCBT and 11 AO) change in rumination was no longer significant (F = 2.27, df = 1, *p* = .15, *η^2^=.11*) and change in depression was reduced to a trend on the CDRS-R (F = 3.80, df = 1, *p* = .07, *η^2^=.16*) and the RADS (4.16, df = 1, *p* = .06, *η^2^=.17*).

*RFCBT Engagement and Change in Rumination*. Adolescent engagement was coded based upon recordings and treatment session notes with the following ratings: 0 = dropped out, 1 = present at sessions, but no homework, 2 = some engagement and partial homework completion, 3 = full engagement and homework completion, 4 = internalized skills. The reliable change index (RCI) was calculated for each participant and the Edwards-Nunally corrected value was used as it adjusts pre-treatment scores for regression to the mean (Speer, 1992). Among the completer sample, fifty-seven percent of adolescents receiving RFCBT achieved reliable change in rumination, whereas 14% of adolescents in the AO group achieved reliable change in rumination. The R-squared for prediction of change in rumination based on engagement was 0.22 as illustrated in Figure S1.

*Brooding and Self-reflection.* Additional MRMs were run on the ITT sample for the brooding and rumination subscales of the RRS. The group by time interaction was significant for brooding (F – 6.13, df = 110, *p* = .02), but not for self-reflection (F = 1.47, df = 111, *p* = .23), suggesting that RFCBT serves to reduce the maladaptive features of rumination.

Supplemental References:

Watkins ER, Mullan E, Wingrove J, Rimes K, Steiner H, et al. (2011) Rumination-focused cognitive-behavioural therapy for residual depression: phase II randomised controlled trial. Br J Psychiatry 199: 317-322.

Speer DC. (1992) Clinically significant change: Jacobson and Truax (1991) revisited. J Consult Clin Psychol 60: 402-408.
